# Lifelong vegetarianism and breast cancer risk: a large multicentre case control study in India

**DOI:** 10.1186/s12905-016-0357-8

**Published:** 2017-01-18

**Authors:** Toral Gathani, Isobel Barnes, Raghib Ali, Rajkumar Arumugham, Raju Chacko, Raghunadharao Digumarti, Parimal Jivarajani, Ravi Kannan, Dasappa Loknatha, Hemant Malhotra, Beela S. Mathew, on behalf of the INDOX Cancer Research Network Collaborators, Raghib Ali, Rajkumar Arumugham, Radhu Ananthakrishnan, Sivensen Balasubramanian, Isobel Barnes, Raju Chacko, Anil D’Cruz, Raghunadharao Digumarti, Gita Doshi, Mary Foulkes, Trivadi Ganesan, Toral Gathani, Sanjay Gupta, Parimal Jivarajani, K Chandramohan, Ravi Kannan, Dasappa Loknatha, Hemant Malhotra, Mohan Mallandas, Beela S. Mathew, Shaesta Mehta, Reena Nair, Paul Sebastian, Atul Sharma

**Affiliations:** 1 Cancer Epidemiology Unit, Nuffield Department of Population Health, University of Oxford, Richard Doll Building, Roosevelt Drive, Oxford, OX3 7LF UK; 2 Oxford University Hospitals NHS Foundation Trust, Oxford, UK; 3 G Kuppuswamy Naidu Memorial Hospital, Coimbatore, India; 4 Christian Medical College, Vellore, India; 5 Nizams Institute of Medical Sciences, Hyderabad, India; 6 Gujarat Cancer Research Institute, Ahmedabad, India; 7 Cachar Cancer Hospital and Research Centre, Silchar, India; 8 Kidwai Memorial Institute of Oncology, Bangalore, India; 9 RK Birla Cancer Centre, SMS Medical College, Jaipur, India; 10 Regional Cancer Centre, Trivandrum, India

**Keywords:** Diet, Breast cancer, India, Risk factors

## Abstract

**Background:**

The lower incidence of breast cancer in Asian populations where the intake of animal products is lower than that of Western populations has led some to suggest that a vegetarian diet might reduce breast cancer risk.

**Methods:**

Between 2011 and 2014 we conducted a multicentre hospital based case—control study in eight cancer centres in India. Eligible cases were women aged 30–70 years, with newly diagnosed invasive breast cancer (ICD10 C50). Controls were frequency matched to the cases by age and region of residence and chosen from the accompanying attendants of the patients with cancer or those patients in the general hospital without cancer. Information about dietary, lifestyle, reproductive and socio-demographic factors were collected using an interviewer administered structured questionnaire. Multivariate logistic regression models were used to estimate the odds ratio (OR) and 95% confidence intervals for the risk of breast cancer in relation to lifelong vegetarianism, adjusting for known risk factors for the disease.

**Results:**

The study included 2101 cases and 2255 controls. The mean age at recruitment was similar in cases (49.7 years (SE 9.7)) and controls (49.8 years (SE 9.1)). About a quarter of the population were lifelong vegetarians and the rates varied significantly by region. On multivariate analysis, with adjustment for known risk factors for the disease, the risk of breast cancer was not decreased in lifelong vegetarians (OR 1.09 (95% CI 0.93-1.29)).

**Conclusions:**

Lifelong exposure to a vegetarian diet appears to have little, if any effect on the risk of breast cancer.

## Background

Breast cancer is the most commonly diagnosed cancer in women globally, with approximately 1.67 million cases in 2012 [1]. The increase in global incidence is largely attributable to rising incidence rates in less developed countries such as India, where breast cancer is now the most commonly diagnosed malignancy in women with over 150 000 incident cases annually [2].

The lower incidence of breast cancer in Asian populations where the intake of animal products is lower than that of Western populations has led some to suggest that a vegetarian diet might reduce breast cancer risk [3].

Diet related factors which have established associations with breast cancer risk include alcohol consumption and obesity [4, 5]. There is little evidence to support a relationship between a vegetarian diet and subsequent breast cancer risk in cohort studies conducted in largely Western populations [6–8]. However some small studies in India and South Asian migrants in the United Kingdom have suggested that a vegetarian diet may be associated with a reduced risk of breast cancer, although the results are inconclusive [9–12].

India provides a unique opportunity to examine the role of diet in relation to cancer risk, as vegetarianism in India is associated with unique characteristics in that it is usually a lifelong pattern and of a much higher prevalence than in the West where most studies examining this association have been set [13]. We report here the findings of a multicentre case control study on the role of lifelong vegetarianism in the aetiology of breast cancer in Indian women, allowing for known risk factors for the disease.

## Methods

Between 2011 and 2014, a multicentre case—control study was conducted in eight participating hospitals in India (Table 1). Ethics approval for the study was obtained at each participating centre. A common protocol was used at all the centres and all consent forms and participant information sheets were available in English and local dialects at each participating centre. Written consent was obtained from each participant.

**Table 1:** Proportion of life long vegetarians (LLVs) in each participating centre (cases and controls combined)

Centre	City	State	Proportion of LLVs (%)
Birla Cancer Centre, SMS College	Jaipur	Rajasthan	74
Gujarat Cancer Research Institute	Ahmedabad	Gujarat	69
Christian Medical College	Vellore	Tamil Nadu	20
G Kuppuswamy Naidy Memorial Hospital	Coimbatore	Tamil Nadu	19
Kidwai Memorial Institute of Medical Sciences	Bangalore	Karnataka	19
Nizam’s Institute of Medical Sciences	Hyderabad	Andhra Pradesh	15
Regional Cancer Centre	Trivandarum	Kerala	8
Cachar Cancer Centre	Silchar	Assam	<1

Eligible cases consisted of women with a histologically confirmed diagnosis of invasive breast cancer (ICD-10 C50) diagnosed within the preceding six months and presenting to the hospital for diagnosis and/or treatment. Patients with a past history of breast cancer were excluded, but patients with a first presentation of metastatic breast cancer were eligible for participation. Two groups of hospital based controls were used: (1) women without a diagnosis of cancer who were accompanying cancer patients and (2) women who had been admitted to the hospital with illnesses not related to the exposure of interest e.g. trauma. Approximately 80% of the controls were accompanying cancer patients with the majority accompanying non breast cancer patients.

All controls were frequency matched to cases by age (30–39, 40–49, 50–59, 60–69 years old) and state of residence (see Table 1). We fixed the minimum sample size required at 1600 for each group to give 80% power to detect an odds ratio of 0.8 at the 5% significance level, assuming the proportion of lifelong vegetarians is 30%.

All interviewers received common training from a single lead principle investigator in how to conduct the interviews and complete the questionnaires. At the participating hospitals, interviewers were trained by a principle investigator to identify potential participants. Information on sociodemographic, dietary lifestyle and reproductive factors were collected using pre-tested interviewer administered structured questionnaires. Interviewers measured the height and weight of all participants. The cases were asked specifically about their weight before illness, as a cancer diagnosis can potentially influence dietary habits and cancer treatments can influence weight loss or gain. A vegetarian diet is defined as a diet that does not contain meat, poultry or fish [13]. A person who was a lifelong vegetarian was therefore defined as someone reporting that not only were they currently not eating meat, poultry or fish but that they had never eaten meat, poultry or fish. Egg consumption was recorded separately and was considered to be part of a vegetarian diet. Socioeconomic status was assessed from questions that related to location and type of housing, and facilities available within housing, such as running water.

### Data analyses

A logistic regression model was used to examine the association between risk of breast cancer and lifelong vegetarianism. The model was stratified by age (in 10 year age groups) and state of residence, to account for frequency matching, and adjusted for known risk factors. These included body mass index (BMI) before illness (<19, 19–25,25–20,>30 kg/m^2^); the interaction between parity and duration of breast feeding (0–3/<4, 0–3/4+, 4+/<4, 4+/4+ children/years); as well as self-reported location of house (urban/rural) and the presence of running water in the house (no/yes), which were used as indicators of socioeconomic status.

Odds ratios (OR) and 95% confidence intervals (CI) are reported. Women with missing data for lifelong vegetarianism (1.1%) were excluded from the analysis. Women with missing values for a given adjustment variable (8.0% for BMI; 7.2% for the interaction between parity and breast feeding; 0.8% for location of house; and 0.8% for running water) were assigned to a separate category for that variable, but these data are not shown. Statistical tests of heterogeneity across categories of a variable were performed using likelihood ratio tests.

In this study, 33% of the controls reported to be accompanying a patient with breast cancer although they may not have necessarily been residing in the same dwelling. However, this might bias our results as individuals from the same household may share similar dietary patterns. To assess this possibility we performed a sensitivity analysis restricting the controls to those who did not report accompanying a patient with breast cancer.

All data was entered centrally onto a custom designed database with internal validity checks. All analyses were performed at the University of Oxford using Stata V12.

## Results

The study population consisted of 2 101 cases and 2 255 controls in the study and the characteristics of the study population are summarised in Table 2.

**Table 2:** Characteristics of the study population

	Case	Control
	(*N* = 2 101)	(*N* = 2 255)
Sociodemographic variables		
Age in years at recruitment, mean (SD)	49.7 (9.7)	49.8 (9.1)
Hindu religion, % (N)	83 (1 741)	79 (1 790)
Living in urban location, % (N)	58 (1 223)	50 (1 124)
Running water in house, % (N)	84 (1 762)	78 (1 732)
Living in stone house, % (N)	88 (1 836)	84 (1 881)
Self-contained toilet in house, % (N)	88 (1 848)	88 (1 959)
Housewife, % (N)	73 (1 531)	70 (1 562)
Diet, alcohol and tobacco		
Life long vegetarianism, % (N)	29 (611)	25 (560)
Ever consumed alcohol, % (N)	<1 (13)	<1 (12)
Ever smoked tobacco, % (N)	1 (11)	2 (35)
Ever chewed tobacco, % (N)	6 (133)	10 (231)
Ever chewed non-tobacco, % (N)	9 (193)	16 (358)
Anthropometry		
Body Mass Index in kg/m^2^, mean (SD)	25.4 (9.0)	24.6 (7.1)
Height in cm, mean (SD)	154.5 (7.7)	154.0 (7.9)
Reproductive factors		
Age in years at menarche, mean (SD)	13.8 (1.3)	13.7 (1.3)
Age in years at first birth, mean (SD)	22.8 (5.3)	22.3 (4.7)
Number of living children, mean (SD)	2.5 (1.2)	2.6 (1.2)
Total duration of breast feeding (parous women), mean (SD)	4.1 (2.4)	4.6 (2.7)
Postmenopausal at recruitment, % (N)	56 (1 166)	55 (1 204)
Ever used oral contraceptive pill, % (N)	3 (62)	2 (51)
Ever used hormone replacement therapy, % (N)	<1 (3)	<1 (3)
Sterilised, % (N)	30 (635)	37 (821)

The mean age at recruitment into the study was similar in cases and controls (49.7 years versus 49.8 years). Approximately three quarters of the study population were housewives and four out of five participants were of Hindu religion. A higher proportion of the cases lived in a town or city compared to the controls (58% versus 50%), had running water in their property (84% versus 78%) and lived in a stone rather than mud house (88% versus 84%). Eighty-eight percent of both cases and controls reported having a self-contained toilet within their property.

The overall proportion of lifelong vegetarians in the study was 29% of cases (*N* = 611) and 25% of controls (*N* = 560). There was significant geographic variation with rates of over 70% in Rajasthan and Gujarat, and <10% in the north eastern state of Assam and in Kerala. In general among the LLVs, consumption of eggs was extremely low with only 3% (*N* = 31) reporting that they had ever eaten eggs. The consumption of both alcohol and smoking of tobacco was virtually non-existent, but around 10% of the study population chewed tobacco or non-tobacco products.

The mean height of cases and controls was similar (154.5cms versus 154.0cms), but the mean body mass index (BMI) was higher among the cases (25.4 kg/m^2^ versus 24.6 kg/m^2^).

Mean age at menarche was around 14 years and just over half of the study participants reported being postmenopausal at recruitment. Nulliparity and absence of breastfeeding was rare among all women, and the mean number of living children was similar for both cases and controls (2.5 vs 2.6) with mean number of years of breastfeeding of 3.9 years for cases compared to 4.4 years for the controls. The use of exogenous hormones, either oral contraceptives or hormone replacement therapy was virtually non-existent. About a third of the whole study population had undergone a procedure for sterilisation.

**Fig. 1:**
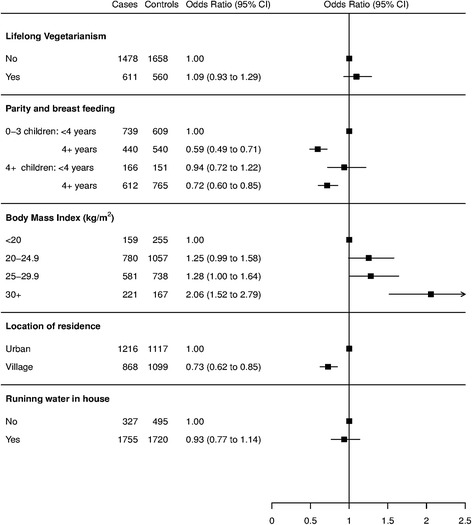
Forest plot showing the adjusted odds ratios with 95% confidence intervals for the association between potential risk factors and breast cancer in women in India: matched by age and geography. The odds ratios were adjusted for diet, parity & breast feeding, BMI before illness, house location and running water

The results of the multivariate analysis are shown in Fig. 1. There was no evidence of any difference in the risk of breast cancer between vegetarians and non-vegetarians (OR 1.09, 95% CI 0.93-1.29). There was evidence that the risk of breast cancer risk was associated with the interaction between parity and breast feeding, BMI, and location of residence. Women, regardless of parity, who had breastfed for 4 or more years had less risk of breast cancer than those who breastfed for less than 4 years (OR 0.59, 95% CI 0.49-0.71 for three or less children and OR 0.72, 95% CI for four or more children).

The risk of breast cancer in obese women with BMI >30 kg/m^2^ was approximately twice that of women with BMI < 20 kg/m^2^ (OR 2.06, 95% CI 1.52-2.78). Women who lived in villages had 27% less risk of breast cancer than those who lived in urban areas (OR 0.73, 95% CI 0.63-0.84). The results of the sensitivity analysis showed that any bias caused by recruitment of attendants accompanying breast cancer patients was likely to be small. The association seen in the restricted analysis was not different to the main result seen in the full analysis for life long vegetarian diet and breast cancer risk (OR 1.13, 95% CI 0.93 - 1.37 versus OR 1.09, 95% CI 0.93-1.29).

## Discussion

In this multicentre case control study in India we find no evidence of an association between exposure to a lifelong vegetarian diet and subsequent risk of breast cancer, after adjusting for known risk factors.

Lifestyle factors, including dietary patterns, have long been recognised as potentially important determinants of cancer risk. The interest in a possible association between diet and risk of breast cancer is stimulated by the observed lower risk of breast cancer in South Asian populations and in South Asian diaspora [2, 9]. There is significant global variation in the prevalence of vegetarianism within countries, and India is known to have one the highest proportion of vegetarians in its population. Furthermore, vegetarianism in India is associated with unique characteristics in that it is usually a lifelong pattern. The overall proportion of lifelong vegetarians of about 30% and the variation in regional rates of lifelong vegetarians in this study are consistent with national data [13–15]. The National Family and Health Survey of India has shown that the highest proportion of vegetarians reside in the states of Rajasthan and Gujarat and that there are over 14 states in India where the rates of vegetarian diet in women is less than 10% including Assam and Kerala, and our patterns are similar [16].

Large prospective studies which have examined the effect of diet on breast cancer risk in Western populations have not shown any significant effect, with the overall risk of breast cancer being largely similar in women who consume either a non-vegetarian or vegetarian diet [6–8, 14]. The results of the present study are in line with results of a recent meta-analysis of prospective cohort studies showing no significant decreased risk of breast cancer in vegetarian compared to non-vegetarian women [17].

Although studies in India and of South Asians residing in the United Kingdom have suggested a possible lower risk of breast cancer in vegetarians, the findings were non-significant as the studies have largely been small and underpowered [9–11]. Our study was appropriately powered to investigate any potential association between lifelong vegetarianism and breast cancer risk, and we find no association with a lifelong vegetarian diet and risk of breast cancer, after adjustment for known risk factors for the disease.

Overweight and obese postmenopausal women are at an increased risk of breast cancer compared to women of normal weight in Europe [5, 18, 19] and in India [20, 21]. In our study, the effect of raised BMI on breast cancer risk was clear, with obese women at a higher risk of breast cancer compared to women with BMI of less than 20 kg/m^2^ (OR 2.06). India is currently facing a challenging public health problem regarding malnutrition, as rising rates of obesity are being seen in urban populations whilst undernutrition remains a challenge in rural parts of the country, and there is significant heterogeneity within the country of under and over nutrition. The mean BMI of the participants in this study was over 25, and probably reflective of the participating centres. Three of our participating centres are in the states of Tamil Nadu and Kerala, where rates of women being overweight or obese are among the highest in India, and none of our participating centres are in states where undernutrition is very high [15, 16].

Other lifestyle factors which are known to raise breast cancer risk in Western women include the consumption of alcohol [4] and use of exogenous hormones in the form of hormone replacement therapy [22] and the oral contraceptive pill [23] and exposure to these risk factors was very low in this population. Low consumption of alcohol and smoking tobacco has been reported previously [24, 25] but there are little published data on use of HRT among Indian women. Use of chewable tobacco and non-tobacco products was slightly higher and although a small study from the north east India has suggested an increased risk of breast cancer in women who chew betel nut, we find no such association [25].

The single most important reproductive factor associated with breast cancer risk in this study was breastfeeding and substantial reductions in breast cancer risk were seen in women who engaged in longer durations of breast feeding, regardless of parity. The protective effect of breastfeeding on breast cancer risk is established, with approximately a 4% reduction in breast cancer risk per 12 months of breastfeeding and no significant differences are seen in the magnitude of the reduction in risk in women in developed and developing countries [26].

The main strength of this study is that it was powered to examine the relationship between diet and breast cancer risk and the final numbers recruited were adequate to reliably examine the association, and to our knowledge this is the largest study conducted in India to investigate this association. Detailed information about known risk factors for breast cancer was also collected to enable appropriate adjustments to be made, and by comparing the distribution of these known risk factors to nationally available data, we have shown that the study population is representative. The main limitation of this work is that case controls studies are subject to recall bias and examining the relationship between diet and cancer risk using this study design is challenging. A cancer diagnosis may influence the dietary and lifestyle patterns of a patient. This should not be an issue for life-long vegetarians, but to minimise this source of bias we restricted recruitment to cases who had been diagnosed with breast cancer within the previous six months.

## Conclusions

We find that exposure to a lifelong vegetarian diet appears to have little, if any effect on breast cancer risk but established risk factors such as breastfeeding and body mass index are very important. The rising incidence rates of breast cancer in developing countries is a public health concern, and findings from studies such as these emphasise the importance of tackling public health challenges such as obesity, in order to prevent some of the growing breast cancer burden.
